# Effects of study area size on geographic characterizations of health events: Prostate cancer incidence in Southern New England, USA, 1994–1998

**DOI:** 10.1186/1476-072X-5-8

**Published:** 2006-02-15

**Authors:** David I Gregorio, Holly Samociuk, Laurie DeChello, Helen Swede

**Affiliations:** 1 Department of Community Medicine & Health Care, University of Connecticut School of Medicine, 263 Farmington Ave., Farmington, CT, 06030-6205, USA; 2 Connecticut State Department of Public Health, Hartford CT, USA

## Abstract

**Background:**

We consider how representations of geographic variation in prostate cancer incidence across Southern New England, USA may be affected by selection of study area and/or properties of the statistical analysis.

**Method:**

A spatial scan statistic was used to monitor geographic variation among 35,167 incident prostate cancer cases diagnosed in Massachusetts, Connecticut and Rhode Island from 1994 to 1998, in relation to the 1990 populations of men 20+ years of age living in that region. Results from the combined-states analysis were compared to those from single-states. Impact of scanning procedures set to examine up to 50% or no more than10% of at-risk populations also was evaluated.

**Results:**

With scanning set to 50%, 5 locations in the combined-states analysis were identified with markedly distinct incidence rates. Fewer than expected cases were estimated for nearly all Connecticut, Rhode Island and West Central Massachusetts, whereas census tracts on and around Cape Cod, and areas of Southwestern Connecticut and adjacent to greater Boston were estimated to have yielded more than expected incidence. Results of single-state analyses exhibited several discrepancies from the combined-states analysis. More conservative scanning found many more locations with varying incidence, but discrepancies between the combined- and single-state analysis were fewer.

**Conclusion:**

It is important to acknowledge the conditional nature of spatial analyses and carefully consider whether a true cluster of events is identified or artifact stemming from selection of study area size and/or scanning properties.

## Background

Spatial analyses of health events can provide helpful information that informs our understanding of the determinants and control of diseases within populations. Geographic studies have discerned the non-random nature of health hazards [1], at-risk populations [2], disease occurrence [3,4], progression [5], screening [6], treatment [7,8], and end results [9,10].

By their nature, spatial analyses of health events are zero sum problems wherein overall rates (cases per 100,000 persons), proportions (deaths among diagnosed cases) or case counts (birds carrying West Nile virus) applicable to a defined study area (nation, region, state) are disaggregated to measure relative differences across smaller analytic units (counties, census tracts, exact coordinates). Changing event counts and/or underlying populations by expanding or contracting study area size and/or modifying properties of the spatial statistic can affect estimates of spatial variation therein. Similarly, the sensitivity of the spatial statistic to identify event clusters specific to any given location is known to vary by changing the proximity of that location to a study area's boundaries [11]. As such, findings for any given spatial analysis are best considered as conditional and modifiable as a consequence of study area size and/or properties of statistical procedures.

Nationwide efforts to foster regional health information networks/organizations (RHINs/RHIOs) that span traditional geo-political boundaries demand greater understanding of how aggregating health and population data may affect analysis and interpretation of disease patterns. To date, there are few opportunities to evaluate consequences of study area size selection. Sharing health related data across states or regions is uncommon, if not restricted, in the interest of protecting individual privacy and confidentiality of information. Moreover, inconsistencies across states regarding their use of geocoding references, statistical and mapping software further limit possibilities to pool/combine data for multi-state studies. Hence, researchers have limited guidance as to how (and to what extent) findings may change by modification of the study area.

We previously described provisional qualities of spatial analyses related to geocoding [12], choice of statistical methods [13,14]; surveillance period [15], covariate adjustment [16], and spatial units chosen for analysis [17]. Here, we consider the conditional effect of study area size on the representation of health events. The situation is illustrated using data on prostate cancer incidence in Southern New England, USA. The data reported here are unusual regarding both their level of detail (census tract for cases and population) and coverage (3 contiguous jurisdictions – states). Discerning the potentials and pitfalls of spatial analytic methods will facilitate the dissemination of methods among researchers and practitioners focused on health surveillance and health system evaluation.

## Results

Among the 35,167 geocoded records across Southern New England, an average annual age-adjusted incidence rate of 194.6 incident cases per 100,000 at-risk men was observed (See Table 1). Within Connecticut, the statewide average annual age-adjusted incidence rate of 201.7 prostate cancers per 100,000 at-risk men (5.2% above the composite rate among men outside Connecticut) was noted. Comparable incidence rates were 182.8 for Rhode Island (93% of the rate outside Rhode Island) and 192.6 for Massachusetts (97% of the rate outside Massachusetts).

**Table 1:** Prostate cancer incidence (1994–1998) and population (1990) for Southern New England and single states, USA.

	Reported Cases	Coded Cases (%)	Population at-risk^1^	Avg. Annual incidence^2^	Census tracts
CT-MA-RI	38,956	35,167 (90)	3,606,842	194.6	2,400
Massachusetts (MA)	22,525	20,243 (90)	2,097,746	192.6	1,331
Connecticut (CT)	12,501	11,735 (94)	1,160,925	201.7	834
Rhode Island (RI)	3,930	3,189 (81)	348,171	182.8	235

^1 ^1990 U.S. Census of males, 20+ years old. ^2 ^Per 100,000 at-risk persons.

### Combined-states study area

**Figure 1:**
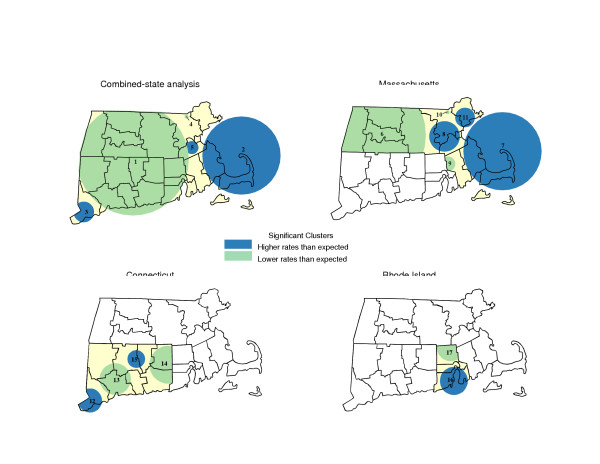
Places of significant variation in invasive prostate cancer incidence rates (using the default scanning window of up to 50%) for Southern New England and its constituent states, USA, 1994–1998.

Our first comparison of combined and single-state analyses employs the spatial scan statistic with properties set to evaluate rates within locations containing as much as 50% of a study area's at-risk population (1.8 million men in the combined-states study area, 580 K within Connecticut, 174 K within Rhode Island and 1.05 million within Massachusetts). Rate variation was observed across Southern New England regardless of the size of the study area (See Figure 1 and Table 2).

**Table 2:** Geographic variation of prostate cancer incidence using the default (50%) scanning window according to selected study areas across Southern New England, USA, 1994–1998.

Study Area	Location*	Population at-risk	Size (sq km)	Cases	O/E**	p-value
CT, MA & RI	1	1,779,267	28,174.1	16,055	0.91	0.0001
	2	200,603	13,809.5	2,729	1.27	0.0001
	3	115,636	973.1	1,626	1.33	0.0001
	4	31,229	36.1	127	0.49	0.0001
	5	63,951	382.9	830	1.26	0.0001
						
Massachusetts (MA)	6	408,741	37,868.3	3,465	0.81	0.0001
	7	200,603	13,809.5	2,729	1.26	0.0001
	8	518,190	2,102.5	5,076	1.15	0.0001
	9	29,381	497.2	114	0.45	0.0001
	10	31,229	36.1	127	0.48	0.0001
	11	177,096	865.7	2,194	1.20	0.0001
						
Connecticut (CT)	12	140,890	1,313.8	1,897	1.28	0.0001
	13	353,654	2,249.7	3,144	0.86	0.0001
	14	64,714	3,261.4	433	0.69	0.0001
	15	116,216	713.5	1,293	1.22	0.0001
						
Rhode Island (RI)	16	66,477	1,794.5	721	1.39	0.0001
	17	168,340	1,699.7	1,366	0.87	0.0001

* Illustrated in Figure 1. ** Risk of prostate cancer for residents of a given location, relative to the risk elsewhere around the designated study area.

When data regarding Connecticut, Rhode Island and Massachusetts were examined simultaneously, 5 locations were identified as having incidence rates likely to differ significantly (p < 0.05) from elsewhere across the combined-states study area. For most of Rhode Island, Connecticut and West Central Massachusetts (Area 1) the age-adjusted average annual incidence rate was estimated to be 91% of expectation relative to the rate among men living elsewhere within the Southern New England region. For a considerably more circumscribed location north of Greater Boston (Area 4), the incidence rate was estimated to be only 49% of the rate observed elsewhere around the combined-states study area.

By comparison, census tracts on and around Cape Cod (Area 2) were estimated to have had an incidence rate 27% greater than other locations within the combined-states study area. Tracts in Southwestern Connecticut (Area 3) and those to the immediate southwest of Greater Boston (Area 5) were observed to have rates estimated to be 33% and 26% higher, respectively, than rates found elsewhere within the study area.

### Single-state study areas

While much consistency between the combined and single-state analyses was evident, several important differences were evident. When considering Massachusetts by itself, for example, 6 locations were identified with rates that differed markedly from elsewhere around the State. Consistent with earlier results, census tracts in the western half of the State (Area 6) yielded an age-adjusted average annual incidence rate 81% of that observed outside that location. Additional locations with markedly low incidence again were found along the Massachusetts borders with Rhode Island (Area 9) and New Hampshire (Area 10).

As in the combined-states analysis, the most likely location of elevated cancer incidence specific to Massachusetts was found among census tracts on and around Cape Cod (Area 7) where the incidence rate was estimated to be 1.26-times greater than expectation. Census tracts around greater Boston (Area 8) revealed a significantly high rate of disease (1.15-times greater than expectation) that spatially encompassed considerably more area, cases and persons at-risk than previously detected within Area 5. Equally noteworthy, the state-specific analysis yielded a location north east of the city (Area 11), with a significantly elevated incidence rate (RR = 1.20) that previously was not identified by the combined-states analysis. Whether this location merits specific attention for disease control efforts depends on which study area is selected for analysis.

Rates specific to Connecticut includes 4 locations that differed from the statewide pattern and finding based on the combined-states study area. Census tracts of Southwestern Connecticut (Area 12) were found, as in Area 3, to have greater than expected incidence (RR = 1.28 in relation, this time, to the rate elsewhere around Connecticut). Whereas the combined-states analysis found the bulk of census tracts across the state to have a lower than expected incidence rate (Area 1), the single-state analysis identified much of the state as having had rates that were not remarkably different from the statewide experience. Here, lower than expected rates were limited to at-risk persons living around West Central Connecticut (Area 13) and the eastern most portions of the State (Area 14). A potential concentration of greater than expected incidence that went unrecognized in the combined-states analysis was a location in North Central Connecticut (Area 15) that included more than 116,000 at-risk men to have had an incidence rate 1.22-times greater than expectation.

The most noticeable disparity between analysis of combined and single-state study areas pertained to Rhode Island where the combined-states analysis suggested nearly all at-risk men were at lower than expected risk of disease. Subsequently in the single-state analysis, however, incidence rates across much of the state appear to have been at or above the overall statewide rate of disease. Here, only among at-risk men living within census tracts along the State's northern border (Area 17) was it estimated that prostate cancer occurred at a rate below that (87%) of what occurred elsewhere around the Rhode Island. In the single-state analysis, men living in South Central census tracts situated around Narragansett Bay (Area 16) were found to have experienced an average annual age-adjusted incidence rate 1.39-times greater than expectation.

### Use of a more restrictive scanning setting

The problem of edge effects and selection of study area can be overcome, to a degree, by modifying properties of the spatial statistic. By rejecting the default SaTScan settings and limiting the spatial scan procedure to include a smaller portion of a study area's at-risk population it is possible to reduce the likelihood that identified clusters will reach or span single-state boundaries. To illustrate, we compared results of combined and single-state analyses when the size of the spatial scan was limited to include no more than 10% of a study area's at-risk population (361 K for the combined-states analysis, 116 K within Connecticut, 35 K within Rhode Island and 210 K within Massachusetts).

**Figure 2:**
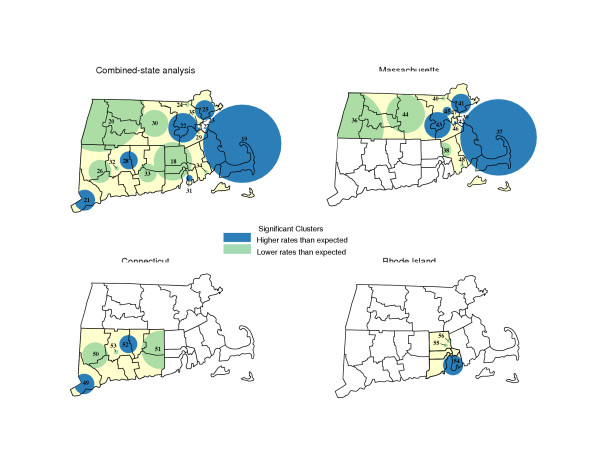
Places of significant variation in invasive prostate cancer incidence rates (using a restrictive scanning window of up to 10%) for Southern New England and its constituent states, USA, 1994–1998.

Predictably, limit on scanning properties yielded more (but smaller) places of likely rate variation. Using the default setting produced 5 significant locations for the combined-states analysis, along with 6, 4 and 2 significant locations for the respective single-state studies, whereas the more restrictive scanning setting yielded 18, 13, 5 and 3 significant locations, respectively (See Figure 2 and Table 3). High rates based on the combined-states study are, for example, now were found on and around Cape Cod (Area 19), communities proximate to Boston (Areas 22, 25, 29 and 35), Greater Hartford (Area 28) and Southwest Connecticut (Area 21). Lower than expected incidence characterized census tracts of Western and Central Massachusetts (Areas 20 and 30), various portions of Connecticut (Areas 26, 32 and 33), and most of Rhode Island.

**Table 3:** Geographic variation of prostate cancer incidence using of a restrictive (10%) scanning window according to selected study areas across Southern New England, USA, 1994–1998.

**Study Area**	Location*	**Population at-risk**	**Size (sq km)**	**Cases**	O/E**	**p_value**
CT, MA & RI	18	340,925	3,306.1	2763	0.79	0.0001
	19	200,603	13,809.5	2729	1.27	0.0001
	20	239,448	17,572.6	1917	0.77	0.0001
	21	115,636	973.1	1626	1.33	0.0001
	22	376,304	1,935.3	3925	1.16	0.0001
	23	5,741	5.3	0	0.00	0.0001
	24	31,229	36.1	127	0.49	0.0001
	25	177,096	865.7	2194	1.21	0.0001
	26	117,550	1,179.9	966	0.77	0.0001
	27	6,027	0.4	0	0.00	0.0001
	28	116,216	713.5	1293	1.24	0.0001
	29	20,338	12.8	220	1.66	0.0001
	30	91,701	1,626.0	774	0.82	0.0003
	31	17,970	86.9	211	1.50	0.0010
	32	28,139	42.5	228	0.71	0.0016
	33	32,013	779.3	225	0.73	0.0118
	34	19,704	28.8	159	0.69	0.0220
	35	3,526	1.3	42	2.33	0.0479
						
Massachusetts (MA)	36	164,636	13,224.3	1325	0.73	0.0001
	37	185,321	13,167.3	2546	1.27	0.0001
	38	29,381	497.2	114	0.45	0.0001
	39	5,741	5.3	0	0.00	0.0001
	40	31,229	36.1	127	0.48	0.0001
	41	177,096	865.7	2194	1.20	0.0001
	42	6,027	0.4	0	0.00	0.0001
	43	204,367	1,542.7	2174	1.19	0.0001
	44	144,083	3,295.9	1193	0.82	0.0001
	45	39,483	169.3	546	1.37	0.0001
	46	20,338	12.8	220	1.64	0.0001
	47	14,521	7.9	126	1.70	0.0010
	48	19,704	28.8	159	0.69	0.0098
						
Connecticut (CT)	49	105,999	920.8	1487	1.32	0.0001
	50	105,495	1,488.9	860	0.75	0.0001
	51	64,714	3,261.4	433	0.69	0.0001
	52	116,216	713.5	1293	1.22	0.0001
	53	28,139	42.5	228	0.69	0.0002
						
Rhode Island (RI)	54	36,996	912.2	424	1.51	0.0001
	55	31,645	31.8	215	0.71	0.0002
	56	28,593	28.3	198	0.75	0.0245

* Illustrated in Figure 2. ** Risk of prostate cancer for residents of a given location, relative to the risk elsewhere around the designated study area.

Patterns observed in the combined-states analysis generally hold for analyses specific to Massachusetts and Connecticut, but as before, the story regarding Rhode Island changes more notable depending on the study area examined. The combine-state analysis yielded evidence of a localized area along the eastern shore of Narragansett Bay with greater than expected incidence, while the northern portion of the state revealed a lower than expected rate of disease. Analysis specific to Rhode Island, however, identified a larger area presumed to have elevated disease rates, while the remainder of the state (with exception of 2 small pockets of low incidence) exhibited rates consistent with the statewide pattern.

## Discussion

This paper examined prostate cancer incidence in Southern New England, 1994–1998, in order to describe whether and how selection of study areas and/or properties of the statistical method could affect estimates of geographic variation among health events. We found combined- and single-state analyses to share much in common, but several discrepancies were noted between approaches. Our analysis also discerned that the extent of discrepancies between combined- and single-state analyses could be reduced by modifying properties of the statistical analysis; limiting the capacity to scan at-risk population for potential disease clusters, reduced the likelihood that identified clusters would span area boundaries.

In essence, 'artifact' resulting from study area size and selection of scanning properties is inevitable in spatial analysis of health events. Understanding the origins of such 'error' is an important step in effectively utilizing available technologies for better disease control. Data analysts must balance a desire for specificity of location of possible event clusters (using a restrictive scanning window) with practical considerations of needing to draw valid generalizations about patterns across large populations or study areas (using a less restrictive scanning window). Studies suspecting focused clusters and those involving limited geographic area may be suitable for more restrictive scanning windows, whereas exploratory analyses and those involving large geographies (regions, nations) may find such restriction impractical (given the likelihood of identifying a large number of clusters) or ill-advised (given the greater potential for Type II error). The volume and severity of edge effects produced necessarily will vary by such decisions.

It could be that when the reference rate for pooled data exceeded the rate of an individual state, the higher expected rate for the expanded study area reduced the likelihood that particular places would exhibit rates that were significantly higher than that new expectation, whereas the likelihood observing places where rates were significantly below that expectation would have been somewhat greater. In most but not all findings reported in Tables 2 and 3, the changes in estimated ratios of observed-to-expected incidence for specific locales were consistent with the modification of the baseline rates from the combined to the single-state study areas. Most illustrative of were changes related to Rhode Island data.

Here, we considered geographic variation in cancer incidence across three jurisdictions (states) that are distinguishable regarding their respective social, political, economic, health care and environmental systems and for which data furnished from three independent registries that may have differed regarding definitions, techniques and standards for reporting cancer incidence. Hopefully, the analysis, unusual for its detail (census tract data) and coverage (3 states), will foster greater appreciation of the opportunities and challenges of pooling health data from contiguous jurisdictions.

## Conclusion

Fully distinguishing 'real' variation due to the geographic distribution of risk, rather than artifact attributable to study area, statistical procedures and/or data systems may not be possible. How particular findings might differ by adding/deleting adjacent areal units to a study area is not typically considered by investigators. Consequently, spatial analysis results may be rightfully considered conditional upon the particular geography selected for study. Unlike epidemiology studies of disease etiology or clinical effect where sampling assures cases are representative of an underlying population, geographic studies of health events do not similarly sample locations within a 'population' of possible places for study, but rather, rely on contiguous areas often aggregated by administrative/political reasons. Short of analyzing entire geographies, there are no *a priori *ways to distinguish the appropriate size or location of study area. Decisions typically rest on suspicion/anecdote regarding the uniqueness of settings and/or the availability of data for study. Hence, these findings underscore the conditional nature of spatial analyses and call for careful consideration before asserting 'true' clusters of events are present.

For the future, states and similar jurisdictions must pursue strategies that maximize potential for data to be pooled and analyzed across conventional geopolitical boundaries. Investment in geocoding, reference street files, data systems and GIS software should commit to principles of data sharing at the same time that procedures are implemented to maintain privacy of personal and group information.

## Methods

Southern New England states of Connecticut, Massachusetts and Rhode Island consist of 17,644 square miles spatially organized within 3 States, 27 counties, 559 towns and 2,400 census tracts. It is home to approximately 3.6 million men 20 years of age and older. The geography of cancer incidence during this period was examined in relation to the populations-at risk within census tracts as enumerated by the 1990 U.S. Decennial Census of the Population, broken down according to ten-year age categories (i.e., 20–29 years, 30–39, 40–49, 50–59, 60–69, 70–79, 80+) [18]. Between 1994 and 1998, a total of 38,956 incident invasive prostate cancers (ICD-9-CM code # 185) were recorded by statewide tumor registries in Massachusetts, Connecticut and Rhode Island. For 35,167 records (90%), the census tract of residence at the time of diagnosis was known and successfully assigned geographic coordinates for analysis; 3,789 records lacked sufficient information to assign a census tract and therefore were excluded from further analysis. The proportion of records assigned census tract locations was substantially greater for Connecticut (94%) and Massachusetts (90%) than Rhode Island (81%). Excluded records typically contained no, incomplete or ambiguous street addresses or addresses that cited P.O. Boxes in place of street addresses. Reason for differences across states was not readily discerned. Previous work suggests that failure to geocode cancer events somewhat under-represented cases among urban dwellers [12].

Based on records available for study, variation in average annual age-adjusted incidence rates across census tracts was evaluated using a spatial scan statistic [19]. The procedure utilizes a large number of scanning circles (>100 K) of varying size and location to search for places (1 or more census tracts independent of conventional geo-political boundaries) where the number of observed cases deviated from a null hypothesis that incidence was proportional to population density (random). Age-adjusted case counts and disease rates within and outside particular circles were determined by ***SaTScan 5.1 ***software [20].

The spatial scan statistic is well suited for disease surveillance, as it does not require a priori assumptions about the number, place or size of locations or direction of effect that may be identified. It takes into account the uneven geographic distribution of the population at risk and, as required, accounts for any number of possible confounding variables. The significance of identified clusters is evaluated using Monte Carlo procedures, with adjusted p-values for multiple testing, to designate locations (clusters of census tracts) where incidence varied from the null. Results of the spatial scan statistic are considered to be conservative estimates of the likelihood of observing events within given locations, relative to places elsewhere around the study area [21].

Spatial analyses of prostate cancer incidence were completed for the 3 state study area of Southern New England, along with analyses specific to Connecticut, Rhode Island or Massachusetts. Findings of significantly high or low concentrations of incident cases are reported in Tables 2 and 3 and illustrated, using Maptitude^® ^software [22], in Figures 1 and 2.

## Competing interests

The authors have not received reimbursements, fees, funding or salary from an organization that may in any way gain or lose financially from the publication of this manuscript, nor do they hold stocks or shares in an organization or other competing financial interests that may in any way gain or lose financially from the publication of this manuscript

## Authors' contributions

D Gregorio conceived of the study and supervised all aspects of its implementation. H Samociuk and L DeChello assisted with the study and completed the analyses. H Swede assisted in interpretation of findings and manuscript preparation.
